# Reduced MHC Class I and II Expression in HPV−Negative vs. HPV−Positive Cervical Cancers

**DOI:** 10.3390/cells11233911

**Published:** 2022-12-03

**Authors:** Andris M. Evans, Mikhail Salnikov, Tanner M. Tessier, Joe S. Mymryk

**Affiliations:** 1 Department of Microbiology and Immunology, The University of Western Ontario, London, ON N6A 3K7, Canada; 2 Department of Oncology, The University of Western Ontario, London, ON N6A 5W9, Canada; 3 London Regional Cancer Program, Lawson Health Research Institute, London, ON N6A 5W9, Canada; 4 Department of Otolaryngology, The University of Western Ontario, London, ON N6A 5W9, Canada

**Keywords:** human papillomavirus (HPV), cervical cancer (CC), major histocompatibility complex (MHC), The Cancer Genome Atlas (TCGA), gene expression, T cell

## Abstract

Cervical cancer (CC) is the second most common cancer in women worldwide and the fourth leading cause of cancer-associated death in women. Although human papillomavirus (HPV) infection is associated with nearly all CC, it has recently become clear that HPV−negative (HPV−) CC represents a distinct disease phenotype with increased mortality. HPV−positive (HPV+) and HPV− CC demonstrate different molecular pathology, prognosis, and response to treatment. Furthermore, CC caused by HPV α9 types (HPV16-like) often have better outcomes than those caused by HPV α7 types (HPV18-like). This study systematically and comprehensively compared the expression of genes involved in major histocompatibility complex (MHC) class I and II presentation within CC caused by HPV α9 types, HPV α7 types, and HPV− CC. We observed increased expression of MHC class I and II classical and non-classical genes in HPV+ CC and overall higher expression of genes involved in their antigen loading and presentation apparatus as well as transcriptional regulation. Increased expression of MHC I-related genes differs from previous studies using cell culture models. These findings identify crucial differences between antigen presentation within the tumor immune microenvironments of HPV+ and HPV− CC, as well as modest differences between HPV α9 and α7 CC. These differences may contribute to the altered patient outcomes and responses to immunotherapy observed between these distinct cancers.

## 1. Introduction

Cervical cancer (CC) is the second most common cancer in women worldwide, and the fourth leading cause of cancer death in women, with an estimated 604,000 new cases and 342,000 deaths worldwide in 2022 [1]. CC arises from normal cervical epithelium through the progressive development of low and high-grade cervical intraepithelial lesions. A major driver for the development of CC is human papillomavirus (HPV) infection which is associated with approximately 85–90% of all CC [2,3]. As opposed to HPV−induced, or HPV−positive (HPV+) CC, no clear etiology has yet been identified for HPV−negative (HPV−) CC, also referred to as HPV−undetected or HPV−independent CC [4].

HPV is a double-stranded DNA tumor virus known to cause cancers of the anus, oropharynx, penis, and cervix [5]. HPV is an epitheliotropic virus that enters through small abrasions to infect the basal layer of the cutaneous and mucosal epithelium [6]. As HPV−infected basal epithelial cells differentiate, they are forced to continuously replicate by the virus, causing the formation of a lesion or papilloma [7]. Although these lesions are often benign, infections with high-risk types of HPV can oncogenically transform cells, promoting the development of cancer. Of the various oncogenic HPV types, HPV16 is the most predominant, accounting for almost 60% of all HPV+ CC [2,3]. HPV16 is a member of the α9 species which also includes HPV31, 33, 35, 52, 58, and 67 [8]. Following HPV16, HPV18 and 45 have the second and third highest association with CC, accounting for approximately 10% and 5% of cases, respectively [2]. HPV18 and 45 are members of the α7 species of viruses, which also includes HPV39, 59, 68, 70, 85, and 97 [8]. Patient outcomes for HPV16+ CC appear to be superior to those of HPV18+ CC [9,10,11,12]. Divergence in the sequence and molecular function of the oncogenes encoded by the different HPV types may contribute to the disparities in patient outcomes by causing differences in the tumor immune microenvironment of these different cancers [13].

The immune response to CC consists of multiple levels of defense that function cooperatively to prevent, control, and eliminate both viral infections and cancer. If the less specific intrinsic and innate immune responses are insufficient to prevent an infection or the development of cancer, subsequent antigen-specific adaptive immune responses will develop in the form of T and B lymphocyte responses [14]. T cell-specific antitumor responses require the presentation of a tumor-associated antigen in the context of major histocompatibility complex (MHC) class I or II [15]. This process is dependent on the initial acquisition of viral or neo-antigenic peptides by antigen-presenting cells (APCs) expressing both MHC class I and II to activate CD8^+^ and CD4^+^ T cells, respectively. Activated CD4^+^ T cells can stimulate the proliferation of CD8^+^ cytotoxic T cells (CTLs) that recognize and respond to their cognate antigenic peptide in the context of MHC class I [16,17]. Interactions of CTLs with neoantigens in the context of MHC class I are critical for the CTL-mediated killing of tumor cells and the anti-cancer immune response [17]. T cell activation, survival, and proliferation require crosslinking of the T cell receptor (TCR) with MHC (signal 1) and the crosslinking of co-stimulatory molecules (signal 2) [15]. Activated CD4^+^ T cells can also interact with B cells to promote the production of antibodies specific to viral antigens and neoantigens. These antibodies will help in viral neutralization, blocking the spread of infection, and preventing the development and spread of cancer [15].

The oncogenes of high-risk HPVs are retained and expressed in HPV−induced cancers and their expression may have effects on the tumor microenvironment (TME). The HPV E6 and E7 oncoproteins have multiple functions within a host cell and are best known for their ability to degrade and inactivate the tumor-suppressors p53 and retinoblastoma protein (pRb), respectively, leading to dysregulation of the cell cycle and accumulation of DNA damage [18,19]. The HPV oncoproteins also contribute to the evasion of the adaptive immune response during infection, through the repression of IFN-stimulated gene expression in keratinocytes, the repression of IRF1 transcriptional activity, and the reduction of MHC class I or II expression on the cell surface [20,21,22,23,24]. In contrast to the immune suppressive activities seen in HPV infection, HPV−induced cancers such as head and neck squamous cell carcinomas (HNSCC) often demonstrate increased infiltration and activation of multiple immune cell types within the TME, indicative of an immune-hot phenotype [25,26]. These HPV+ HNSCC tumors also demonstrate higher expression of MHC class I- and II-related genes compared to HPV− tumors [27,28]. We have recently identified higher levels of tumor-infiltrating lymphocytes and other factors indicative of a more immune-hot phenotype in HPV+ CC compared to HPV− CC [29]. These differences between the TME of HPV+ and HPV− CC may have implications for the outcomes of these cancers. Indeed, HPV+ CC patients appear to have more a favorable prognosis [9,30,31] with clinical and pathological distinctions compared to HPV− CC [32,33]. Unfortunately, the low proportion of HPV− CC compared to HPV+ CC makes a statistical comparison of the characteristics of their tumor immune microenvironments difficult, despite the importance of this research.

The aim of our study was to compare the expression of all classical MHC class I and II genes as well as other key genes involved in their regulation, antigen loading, and presentation, and T cell co-stimulation between HPV α9, HPV α7, and HPV− CC. While many components of these pathways have been examined individually, no systematic and comprehensive analysis has yet been conducted. Despite differences in the pathology, prognosis, and clinical outcomes between HPV+ and HPV− CC, to our knowledge, no studies have systematically compared genes involved in both MHC class I and II expression between these distinct cancers of the cervix. Furthermore, limited information exists on potential differences in antigen presentation between HPV α9 and α7 types in CC [34]. Our analysis of over 200 HPV+ CC in comparison with 19 HPV− CC from The Cancer Genome Atlas (TCGA) demonstrates that differences exist between MHC class I and II expression in these two distinct types of cancer. We observed increased expression of virtually all classical and non-classical MHC class I and II genes in HPV+ tumors as compared to their HPV− counterparts. Similarly, genes encoding proteins vital in their respective antigen presentation pathways were also upregulated in HPV+ tumors. Only modest differences in gene expression were observed between HPV α9 and α7 CC. These differences in MHC class I and II expression between HPV+ and HPV− CC may have implications for the prognosis and clinical outcomes of these cancers and suggest that HPV− CC may be less responsive to immunomodulatory therapies, such as immune checkpoint inhibitors.

## 2. Materials and Methods

### 2.1. Sample Collection and Ethics

The publicly available Broad Genome Data Analysis Center’s Firehose server (https://gdac.broadinstitute.org/, accessed on 2 March 2017) was used to download all data from the TCGA. As a result, no ethical approval was needed.

### 2.2. Histology Verification and Tumor Purity

TCGA histology workflow consisted of a review of hematoxylin and eosin (H&E) stained sections by a tissue site pathologist and an independent pathologist prior to sample acceptance [35]. Only cases that met the criteria for primary cervical cancer according to WHO criteria were accepted [36]. Care was taken to verify that the tumors were not endometrial in origin. Additionally, only samples with ≥60% tumor nuclei and ≤20% necrosis were submitted for nucleic acid extraction [35].

### 2.3. Annotation of HPV Status

A total of 297 CC patient tumor datasets were annotated for HPV status based upon comparative analysis of data extracted from multiple previous independent studies of this cohort [35,37,38,39,40]. HPV status in samples infected with multiple HPV types was annotated as the genotype with the highest expression. As reported previously, samples infected with multiple HPV types showed 2 to over 800,000 times the expression of the dominant type compared to the next most dominant type, which allowed for clear distinction [41]. In the infrequent cases of conflicting data between annotations assigned by previous studies, HPV type or HPV status was defined based on the majority consensus (Table S1). Notably, none of these rare differences in reported HPV type impacted HPV species classification. These minor differences in classification may be related to the breadth of reference genomes used for alignments between these different studies. Although explicit details for each of these prior analyses are not provided in each of the publications, Qiu et al. [38] used 143 different HPV reference genomes, while Banister et al. [37] used only 18 reference genomes. Although this does not provide 100% certainty that HPV− CC samples did not contain HPV DNA from an unusual type that was not specifically used in the alignments, the sequence relatedness between HPV types and the use of many divergent HPV reference genomes reduces the possibility of a sample with an unusual HPV type being inadvertently classified as HPV− CC. Reassuringly, of the 19 TCGA CC samples reported by Ruiz et al. [40] to have undetectable tumor HPV DNA and RNA, 18 were also considered HPV− in our study. The remaining sample (TCGA-C5-A7UI) was classified as HPV33+ by all three other studies that previously reported analysis of this sample [37,38,39] and for that reason was annotated as HPV33+ for this study. Patient samples obtained from secondary metastatic lesions or normal control tissues were omitted from our analysis.

### 2.4. mRNA Expression Comparisons and Statistical Analysis

mRNA expression comparisons were analyzed as carried out previously [29,42,43]. Level 3 RNA-Seq by Expectation Maximization (RSEM) normalized Illumina HiSeq RNA expression data for the TCGA CC cohort were downloaded as described above. RSEM normalization allows for direct comparison of mRNA expression across different cancer types within the TCGA [36]. The CC RNA-sequencing (RNA-seq) dataset consists of 278 HPV+ and 19 HPV− samples. Of these, there are 165 HPV16+, 40 HPV18+, 1 HPV30+, 6 HPV31+, 9 HPV33+, 6 HPV35+, 5 HPV39+, 22 HPV45+, 1 HPV51+, 8 HPV52+, 1 HPV56+, 6 HPV58+, 3 HPV59+, 2 HPV68+, 1 HPV69+, 1 HPV70+, and 1 HPV73+ tumor samples. To correlate cellular mRNA expression to HPV status, the dataset was sorted into 200 HPV α9 (HPV16, 31, 33, 35, 52, and 58 types), 73 HPV α7 (HPV18, 39, 45, 59, 68, and 70 types), and 19 HPV− CC samples (Table S1).

Subsequent statistical calculations were performed using the R program’s wilcox.test function with the conf.level parameter set to 0.95. q-values were calculated for each comparison group with a false discovery rate (FDR) of 10%. Box and whisker plots for gene expression were designed using GraphPad Prism v8.0 (GraphPad Software, Inc., San Diego, CA, USA), and assembled into a final form using CorelDRAW (Corel, Ottawa, ON, Canada). For the boxplots, center lines show the medians and box limits indicate the 3rd (25th percentile) and 4th (75th percentile) quartiles. Whiskers extend 1.5 times the interquartile range (IQR), from Q1 (lower whisker) and Q3 (upper whisker).

## 3. Results

### 3.1. HPV+ and HPV− CC Exhibit Strong Differences in MHC Class II Gene Expression

**Figure 1:**
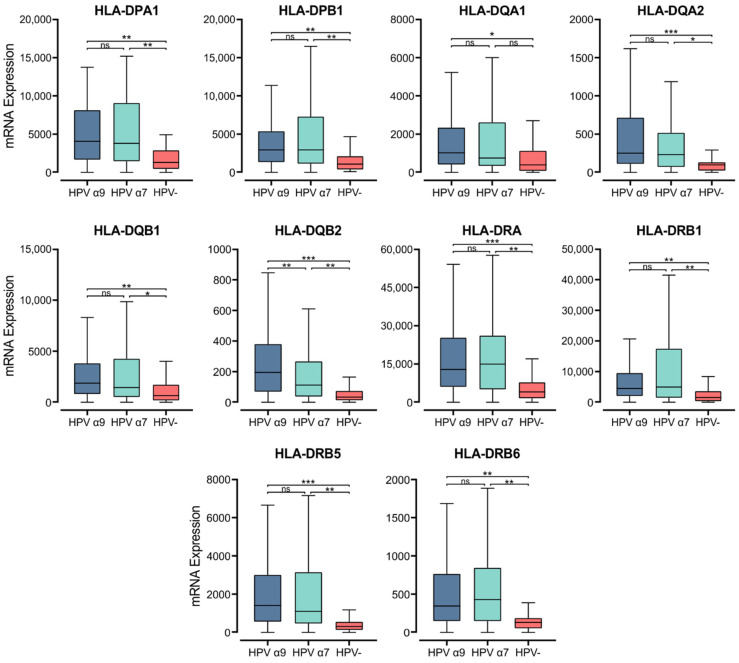
Expression of classical MHC class II α- and β-chain genes in CC stratified by HPV+ (α9 or α7) and HPV− status. Normalized RNA-seq data were extracted from the CC cohort of the TCGA database. *** *p* ≤ 0.001, ** *p* ≤ 0.01, * *p* ≤ 0.05, ns (not significant).

Within the epithelium, MHC class II molecules are classically expressed on professional APCs, including dendritic cells (DC), macrophages, and B cells [44]. Exposure to proinflammatory cytokines can also induce MHC class II expression in cells not considered APCs, including epithelial cells [45]. MHC class II molecules in humans are transmembrane αβ heterodimers with three isotypes: Human Leukocyte Antigen (HLA)-DP, -DQ, and -DR. These are encoded by the α and β chain genes within the HLA locus on chromosome 6 [46]. They present exogenously acquired peptide antigens to stimulate T cell activation [47]. We initially analyzed the normalized Illumina HiSeq RNA expression data from the CC TCGA cohort for the expression of genes encoding the various α- and β- chains for all three classical isotypes. These genes include *HLA-DPA1*, *-DPB1*, *-DQA1*, *-DQA2*, *-DQB1*, *-DQB2*, *-DRA*, -*DRB1*, *-DRB5*, and *-DRB6* (Figure 1). Apart from *HLA-DQA1*, all HPV+ patient samples expressed significantly higher levels of mRNA for the MHC class II classical α- and β-chain genes analyzed versus HPV− tumors. Only for the α9 patient samples was *HLA-DQA1* expression significantly higher. These results indicate that in comparison to HPV− CC, HPV+ CC tumors express elevated levels of the mRNAs encoding the α- and β-chain heterodimers of the classical MHC class II molecules.

Gene expression markers for professional APCs were also examined, specifically *CCL13* (DC), *CD19* (B cells), *CD68*, and *CD163* (Macrophages) (Figure S1). All MHC class II α- and β- chain genes were expressed at levels several orders of magnitude above these markers for professional APCs, except *CD68*. These normalized read levels are also comparable to that of E-cadherin (*CDH1*), an established epithelial cell marker (Figure S1). Therefore, it is likely that these genes are primarily expressed by epithelial cells, and possibly macrophages within the tumor.

### 3.2. Genes Encoding Key Components of the MHC Class II Antigen Presentation Pathway Are Expressed at Higher Levels in HPV+ CC Compared to HPV− CC

**Figure 2:**
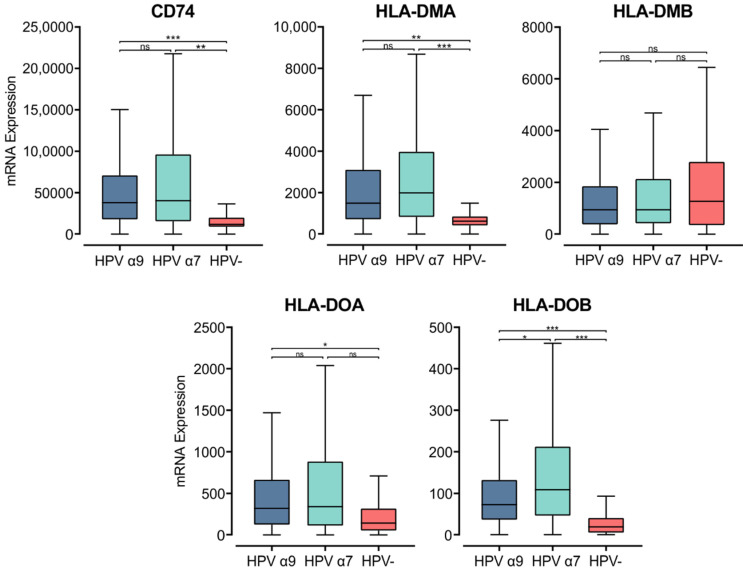
Expression of invariant chain (*CD74*) and MHC class II-like genes in CC stratified by HPV+ (α9 or α7) and HPV− status. Normalized RNA-seq data were extracted from the CC cohort of the TCGA database. *** *p* ≤ 0.001, ** *p* ≤ 0.01, * *p* ≤ 0.05, ns (not significant).

The MHC class II α- and β-chains are synthesized in the endoplasmic reticulum (ER), where they form a trimeric complex with a membrane glycoprotein called the invariant chain (Ii; encoded by *CD74*) to prevent premature loading with endogenously derived peptides [48]. The cytoplasmic region of Ii directs the MHC class II complex through the Golgi and trans-Golgi network to early endosomes. These early endosomes contain cathepsins that proteolytically cleave Ii, leaving a class II-associated Ii peptide in the binding groove (CLIP) [46]. Similar to the genes encoding the MHC class II α- and β-chains, *CD74* was expressed at remarkably high levels and was significantly upregulated in both α7 and α9 HPV+ CC compared to HPV− CC (Figure 2). This finding suggests higher activity of the antigen presentation pathway in HPV+ CC.

CLIP must be removed from the peptide-binding groove for MHC class II to bind antigenic peptides [47,49]. CLIP is released from the peptide-binding groove by HLA-DM, an MHC class II-like heterodimer [50]. HLA-DM is broadly believed to be regulated by another MHC class II-like protein, HLA-DO, although its activity is not fully understood [51]. The genes encoding the α- and β-chains of these heterodimeric class II-like molecules are *HLA-DMA*, *-DMB*, *-DOA*, and *-DOB*. These genes, except for *HLA-DMB* were similarly upregulated in HPV+ CC compared to HPV− (Figure 2). *HLA-DMB* expression trended lower in HPV+ CC compared to HPV−, although this difference was not significant. Furthermore, the *HLA-DMA* and *HLA-DMB* genes are expressed at high relative levels, similar to those for *CDH1* (epithelial cells) and *CD68* (macrophages), suggesting that they may be expressed by these cell types within the tumor (Figure S1). These results further support the above observations, demonstrating consistently increased expression of MHC class II-related genes in HPV+ CC compared to HPV− CC.

### 3.3. Genes That Activate MHC Class II Gene Transcription Are Upregulated in HPV+ CC Compared to HPV− CC

**Figure 3:**
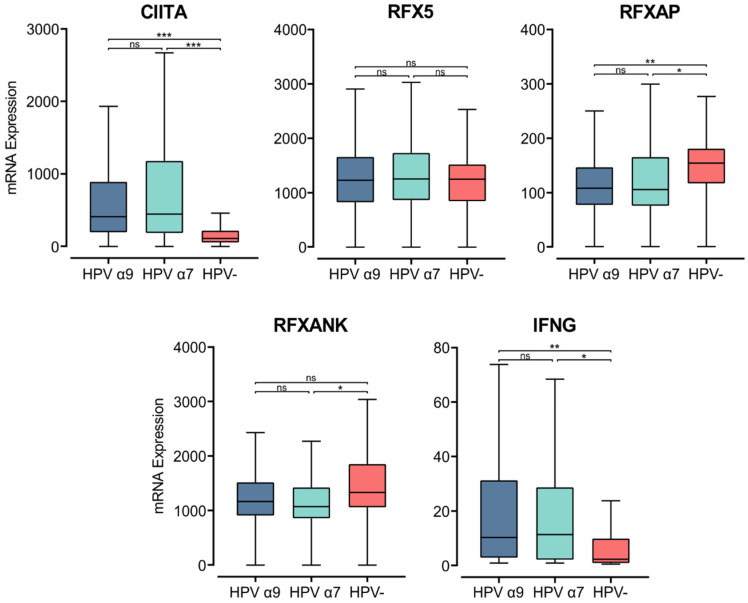
Expression of transcriptional regulators of MHC class II and IFNγ in CC stratified by HPV+ (α9 or α7) and HPV− status. Normalized RNA-seq data for *CIITA*, genes encoding for the RFX family of transcription factors, and IFNγ were extracted from the CC cohort of the TCGA database. *** *p* ≤ 0.001, ** *p* ≤ 0.01, * *p* ≤ 0.05, ns (not significant).

MHC class II transcription is regulated in APCs by the master regulator MHC class II transactivator (*CIITA*) [52]. In accordance with the elevated MHC class II heavy and light chain gene expression, *CIITA* expression was also significantly higher in HPV+ than HPV− tumor samples (Figure 3). The *CIITA* gene is constitutively expressed by professional APCs, including DCs, macrophages, and B cells. Under inflammatory conditions, IFNγ stimulates *CIITA* expression to increase the transcription of MHC class II genes, stimulating antigen presentation by both APCs and non-hematopoietic cells, including epithelial cells [46,52,53]. IFNγ gene (*IFNG*) expression was relatively low, similar in magnitude to that of other leukocyte-specific genes (Figure 3, Supplementary Figure S1). However, *INFG* was expressed at significantly higher levels in HPV+ tumors (Figure 3). The upregulated expression of *CIITA* and *IFNG* in HPV+ CC coordinates with increased expression of the macrophage marker *CD68*, but not with the expression of *CD168* (Figure S1). This association suggests that the increased expression observed in MHC class II-related genes may be a consequence of the exposure of epithelial cells to IFNγ in addition to its expression on macrophages.

CIITA acts as a scaffold within the nucleus, attracting the regulatory factor X (RFX) family of transcription factors to the regulatory regions of MHC-related genes. The heterotrimeric RFX complex formed at MHC class II gene promoters consists of RFX5, RFX-associated protein (RFX-AP), and RFX-ankyrin-containing protein (RFX-ANK) [52,54]. The expression of the *RFX-ANK* and *RFX-AP* genes appears lower in HPV+ compared to HPV− CC, though this difference is only significant for *RFX-AP* (Figure 3). *RFX5* expression appears similar between HPV+ and HPV− CC (Figure 3). These results indicate that expression of the master regulator CIITA may be the rate-limiting factor for the increased expression of MHC class II-related genes in CC.

### 3.4. HPV Status Impacts Expression of T Cell Co-Stimulatory Molecules and Survival Signal Molecules in CC

**Figure 4:**
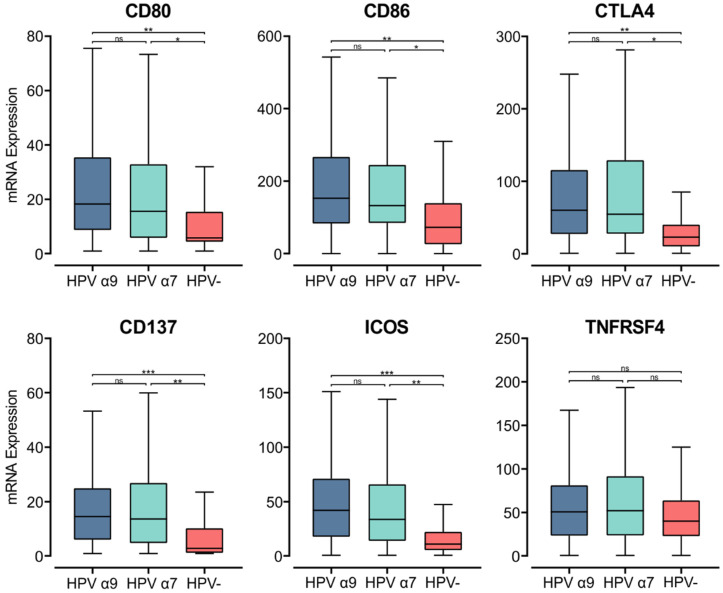
Expression of genes that encode for T cell co-stimulatory and activation molecules in CC stratified by HPV+ (α9 or α7) and HPV− status. Normalized RNA-seq data for genes encoding important costimulatory molecules and T cell activation markers were extracted from the CC cohort of the TCGA database. *** *p* ≤ 0.001, ** *p* ≤ 0.01, * *p* ≤ 0.05, ns (not significant).

Upon the specific recognition of cognate antigenic peptides presented in the context of MHC class II (signal 1), costimulatory molecules are required to trigger TCR signaling and promote T cell activation (signal 2). Interaction of the constitutively expressed CD28 receptor on T cells with either CD80 or CD86 on APCs is the most important costimulatory signal [55]. While *CD28* is constitutively expressed in T cells, *CD80* and *CD86* are induced upon APC activation [56]. In the HPV+ CC cohort, we found significantly higher *CD80/86* mRNA expression compared to their HPV− counterparts (Figure 4). Thus, in HPV+ CC, there is upregulation of MHC class II genes, genes involved in its antigen processing and presentation, and a coordinated upregulation of gene markers associated with APC activation and T cell co-stimulation.

To evaluate relative T cell activation in these tumors, we investigated genes expressed upon T cell activation [56]. We found the mRNA levels of *CD152*, which encodes CTLA-4, were significantly upregulated in HPV+ CC tumors compared to HPV− CC. As CTLA-4 is a marker of T cell activation, its elevated expression indicates increased CD4^+^ T cell activation in the TME of these cancers [56]. Both *CD137* (*TNFRSF9*) and inducible T cell co-stimulator (*ICOS*) were also significantly upregulated in HPV+ CC tumors compared to their HPV− counterparts (Figure 4). *TNFRSF4* (*OX40*, *CD134*) similarly had higher levels of expression in HPV+ CC, although these differences were not significant (Figure 4). *CD137*, *ICOS*, and *TNFRSF4* all encode survival signal molecules that are upregulated in both CD8^+^ and CD4^+^ T cells upon activation by antigen-presenting APCs [56]. Similarly, higher levels of T cells appear to be present in HPV+ CC versus HPV− CC based on T cell markers (*CD3D*, *CD3E*, and *CD3G;* Figure S2). The increased expression of these genes suggests that T cells are being activated by enhanced presentation of their cognate antigens in the context of MHC class II and are subsequently proliferating within the TME of HPV+ CC at higher levels than HPV− CC.

### 3.5. MHC Class I Heavy Chain Genes Are Expressed at Higher Levels in HPV+ Compared to HPV− CC

**Figure 5:**
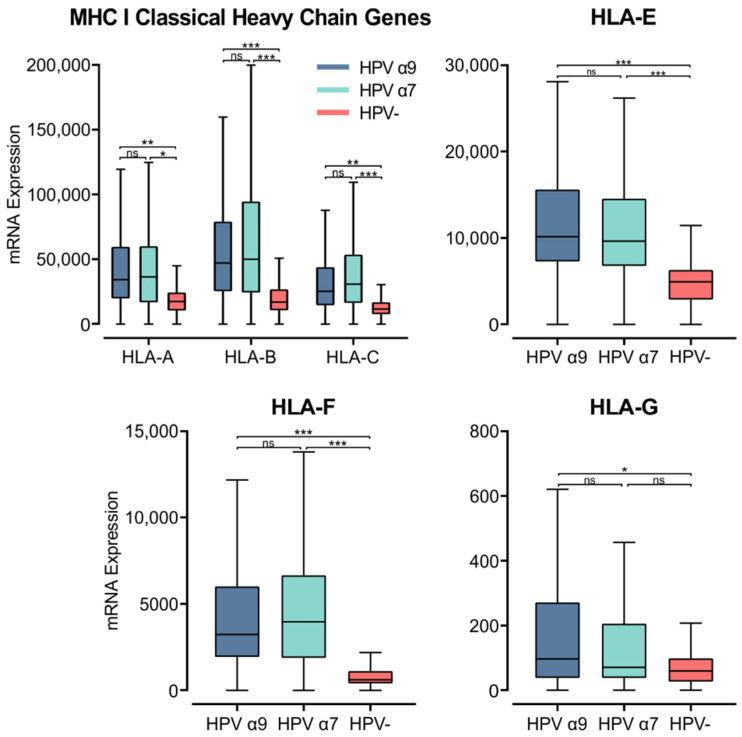
Expression of MHC class I classical and non-classical heavy chain genes in CC stratified by HPV+ (α9 or α7) and HPV− status. Normalized RNA-seq data for MHC class I classical and non-classical were extracted from the CC cohort of the TCGA database. *** *p* ≤ 0.001, ** *p* ≤ 0.01, * *p* ≤ 0.05, ns (not significant).

MHC class I is a heterodimer consisting of a heavy chain and a light chain, that together present a short antigenic peptide [57]. Unlike MHC class II, MHC class I expression is not restricted to APCs and only presents endogenously expressed peptides. However, expressions of both classes of MHC are induced under inflammatory conditions. The heavy chain is encoded by one of three classical (*HLA-A*, *-B*, or *-C*) or non-classical (*HLA-E*, *-F*, or *-G*) genes, while the light chain, or the invariant β_2_-microglobulin, is encoded by the *B2M* gene. Using the Illumina HiSeq RNA expression data from the TCGA CC cohort, we analyzed the expression of the three classical MHC class I genes (Figure 5). All three genes, *HLA-A*, *-B*, and *-C*, demonstrated significantly higher expression in HPV+ CC. These values for gene expression were remarkably high, averaging between 25,000 to 50,000 for HPV+ CC cohorts, in line with the expression of the epithelial marker *CDH1* (Figure S1). Interestingly, the expression of these genes was also higher in the α7 HPV+ CC samples compared to the α9 HPV+ cohort, although these differences were not significant (Figure 5).

Similarly, the non-classical heavy chain genes *HLA-E* and *-F* showed significantly higher expression in HPV+ CC compared to HPV− CC (Figure 5). *HLA-G* only demonstrated significantly higher expression in α9 HPV+ CC compared to HPV− CC (Figure 5). *HLA-G* was also expressed at relatively lower levels overall compared to the other heavy chain genes. As the average mRNA expression for these genes is consistently higher or equivalent in HPV+ samples versus HPV− samples, these results indicate that expression of the HPV oncogenes in actual human CC is not correlated with strong repression of the MHC class I loci as reported in tissue culture-based models [58,59]. The higher levels of MHC class I heavy and light chain expression likely reflect the higher levels of *IFNG* expression present in HPV+ vs. HPV− CC (Figure 3).

### 3.6. HPV Status Affects Components of the MHC Class I Antigen Presentation Apparatus in CC

**Figure 6:**
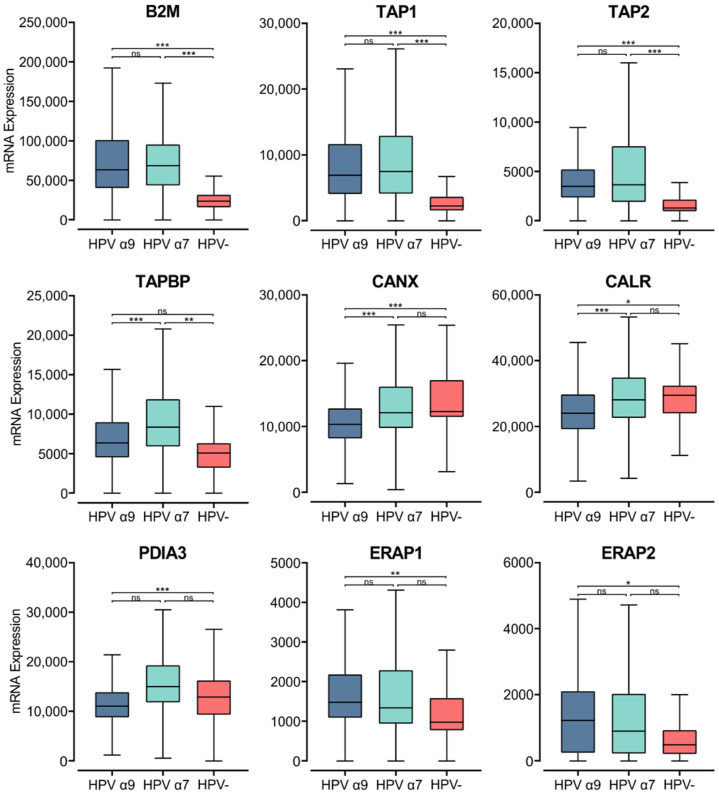
Expression of MHC class I light chain and other genes involved in antigen loading and presentation in CC stratified by HPV+ (α9 or α7) and HPV− status. Normalized RNA-seq data for *B2M* as well as genes important in the MHC class I antigen presentation pathway were extracted from the CC cohort of the TCGA database. *** *p* ≤ 0.001, ** *p* ≤ 0.01, * *p* ≤ 0.05, ns (not significant).

The process of loading peptides to the MHC class I complex requires several accessory proteins with chaperone-like functions [47,60]. Newly synthesized α-chains within the ER bind calnexin, a chaperone protein that retains the MHC class I molecule in a partially folded state. The folding and assembly of a complete MHC class I molecule depends on the association of the α-chain with the β_2_-microglobulin within the ER [47,60]. Analysis of TCGA data revealed high levels of *B2M* transcripts, with significantly lower expression of *B2M* in HPV− CC compared to HPV+ CC (Figure 6).

Upon binding of the β_2_-microglobulin to the α-chain, the partially folded α:β_2_-microglobulin heterodimer dissociates from calnexin, binding a group of proteins called the peptide-loading complex (PLC). The PLC consists of calreticulin, TAP, tapasin, ER aminopeptidase (ERAP), and ERp57. These proteins are integral to maintaining the MHC class I molecule in a state receptive to peptide binding [47,60]. Similar to *B2M*, elevated levels of transcripts were seen in HPV+ CC samples for the genes encoding TAP, calnexin, calreticulin, tapasin, ERAP, and ERp57 (*TAP1/2*, *CANX*, *CANR*, *TAPBP*, *ERAP1/2*, and *PDIA3*, respectively; Figure 6). The *TAP1/2*, *TAPBP*, and *ERAP1/2* genes were all expressed at higher levels in HPV+ CC samples with respect to HPV− CC. Although, for the *ERAP1*/*2* genes, this increased expression was only significant in the HPV α9 cohort. In comparison, *CANX* and *CALR* are expressed at lower levels in HPV+ CC. Interestingly, the expression of *CANX* and *CALR* is significantly lower in α9 HPV+ CC compared to α7 HPV+ CC, with expression in the α7 cohort comparable to that of the HPV− cohort. Similarly, *PDIA3* and *TAPBP* expression are also significantly lower in α9 compared to α7 HPV+ CC. As many genes involved in MHC class I antigen loading and presentation are expressed at higher levels in HPV+ CC, these findings further suggest that MHC class I-dependent presentation of endogenous peptide antigen is occurring at higher levels in these tumors. Again, these findings contrast with decreased TAP transcription reported in tissue culture models [22]. The notable difference in expression of some genes encoding members of the PLC in α9 HPV+ CC compared to α7 HPV+ and HPV− CC indicates that some differences in the regulation of this pathway appear to depend on HPV type.

### 3.7. HPV Status Affects Expression of MHC Class I Transcriptional Regulators

**Figure 7:**
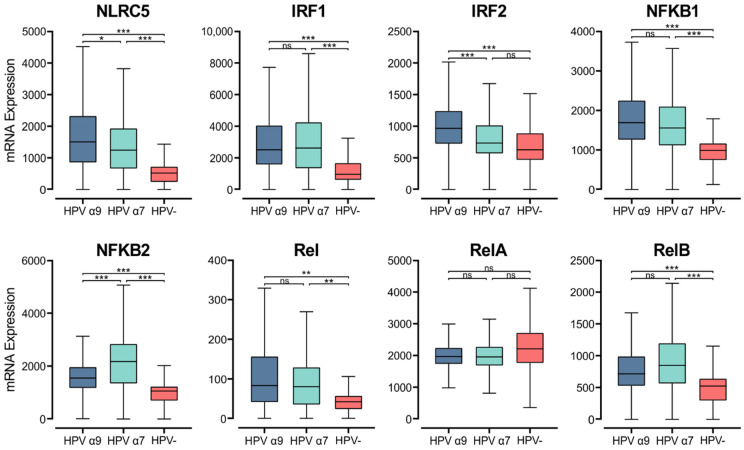
Expression of transcriptional regulators involved in MHC class I expression in CC stratified by HPV+ (α9 or α7) and HPV− status. Normalized RNA-seq data for key transcriptional regulators of MHC class I expression as well as genes in the NF-κB transcription factor family were extracted from the CC cohort of the TCGA database. *** *p* ≤ 0.001, ** *p* ≤ 0.01, * *p* ≤ 0.05, ns (not significant).

MHC class I genes are induced by interferons and transcriptionally controlled by key regulators, although this pathway is not as well understood as it is for MHC class II. NOD-like receptor family CARD domain containing 5 (*NLRC5*), otherwise known as *CITA*, is a key transcriptional activator of MHC class I [61]. Analysis of RNA-seq data demonstrates that *NLRC5* mRNA levels are significantly higher in HPV+ CC than HPV− CC. Furthermore, higher expression of *NLRC5* mRNA is seen in α9 HPV+ CC compared to α7 (Figure 7). The MHC class I enhanceosome formed by NLRC5 also includes the RFX complex, consisting of RFX5, RFX-AP, and RFX-ANK [61]. As described above, *RFX5* expression appears to be similar between HPV+ and HPV− CC, though the expression of *RFXANK* and *RFXAP* is lower in HPV+ CC (Figure 3). These findings indicate that the increased expression of MHC class I-related genes may be primarily caused by increased activity of NLRC5, which may represent the rate-limiting factor.

Upon stimulation by IFNγ, IRF1 and IRF2 are upregulated and can bind the interferon (IFN)-sensitive response element (*ISRE*) motif in the MHC class I promoter region, increasing MHC class I expression [61,62]. In the CC TCGA cohort, both *IRF1* and *IRF2* show higher expression in α9 HPV+ CC compared to their HPV− counterparts (Figure 7). Interestingly, the HPV α7 CC group shows similar *IRF2* expression to that of HPV− CC, and both have significantly lower *IRF2* expression than that in HPV α9 CC.

NF-κB binding to the Enhancer A region is necessary for both the constitutive and inflammation-induced expression of MHC class I [61]. The NF-κB transcription factor family consists of five proteins, RelA, RelB, c-Rel, NF-κB1 (p105/p50), and NF-κB2 (p100/52) that associate with each other to form transcriptionally active homo- and heterodimeric complexes [63]. With the exception of *RelA*, all members of the NF-κB gene family were expressed at significantly higher levels in the HPV+ CC cohorts compared to HPV− CC (Figure 7). We also observed higher expression of gene markers for cells that are the primary producers of IFNγ. Namely, genes encoding for CD3 (*CD3D*, *CD3E*, and *CD3G*) as well as *NKG7* and *CD160*, which encode markers for T and NK cells, respectively (Figure S2). 

Interestingly, HPV+ CC also shows significantly higher levels of apolipoprotein B mRNA editing enzyme catalytic polypeptide *(APOBEC) 3A*, *3B*, and *3H*, with significantly higher expression of *APOBEC3A* and *3B* in α9 CC compared to α7 CC (Figure S3). These APOBEC3 genes are all induced upon IFNγ expression in response to HPV infection, and their genome editing ability is known to increase neoantigen presentation in the context of MHC in these cancers [64,65]. These findings agree with the elevated levels of MHC class I genes and related genes as well as the higher expression of *IFNG* observed in HPV+ CC compared to HPV− CC (Figure 3, Figure 4 and Figure 5).

## 4. Discussion

Despite the well-recognized distinctions between both the pathology [32,33] and clinical outcomes of HPV+ and HPV− CC, few studies have directly compared aspects of the tumor immune microenvironment between these etiologically distinct cancers of the cervix [29]. Furthermore, the small proportion of HPV− CC compared to HPV+ CC limits the statistical power of tests used to compare them. As differences in antigen presentation control T cell-mediated immune responses, changes in their expression have clear implications in both the prognosis and treatment of CC. For this reason, we undertook a systematic analysis of the differences between MHC class I and class II expression in HPV+ and HPV− CC. Our comparison of RNA-seq data for genes involved in both the MHC class I and MHC class II pathways identified an overall trend in increased expression of these genes in HPV+ CC compared to HPV− CC. This likely reflects the “hotter” TME of HPV+ CC since, like other virus-induced cancers, they exhibit increased levels of IFNγ, as well as higher MHC class I and II expression [25,27,28,29,66,67,68].

Limitations of HPV−testing methods may allow for false negatives within the HPV− CC cohort, although these possibilities have been minimized within the TCGA dataset. As all samples within the TCGA CC cohort are carcinomas, it is possible that in a sample with no detectable HPV DNA, HPV contributed to carcinogenesis via a hit-and-run mechanism where the basal cells are permanently altered in pre-cancerous lesions, but HPV DNA is lost in the carcinoma [69]. This may affect gene expression within the HPV− CC cohort and would require investigation of pre-cancerous lesions to confirm the presence or absence of HPV DNA. As low-risk strains of HPV are less prevalent in reference genomes, it may also be possible that CC caused by relatively low-risk types were classified as HPV−. Although, the large number and variety of reference genomes used for HPV typing help to reduce this possibility [37,38]. Furthermore, it has been previously shown that endometrial cancers have been misclassified as HPV− CC, which requires histological analysis to determine the origin of the tumor [70,71]. Although the TCGA reports that great care was taken by study pathologists to verify that the tumors included were not endometrial in origin [35], it remains possible that some were miscategorized. HPV−negativity is more commonly observed in adenocarcinomas rather than squamous cell carcinomas [30] and some of the differences we observe may be related to enrichment for cervical adenocarcinomas [37,38].

While HPV+ CC is commonly caused by several distinct HPV types, HPV16 and 18 are the two most common [10]. These different HPV types exhibit prognostic and molecular differences for patients with HPV+ CC [9,10,11,12]. Although the cumulative risk of cervical intraepithelial neoplasia (CIN) 3+ is much greater for HPV16+ than HPV18+ lesions, HPV16+ tumors are generally associated with favorable overall survival and prognosis [9,10,11]. Furthermore, studies have concluded that HPV16+ tumors are more sensitive to radiotherapy and chemoradiotherapy than HPV18+ tumors, leading to better prognosis [9,12]. Although there are several proposed reasons for the differing prognosis and survival between CC caused by different HPV genotypes, such as their different frequencies of integration, the mechanism remains largely unknown [35,72].

Given the key role of antigen presentation in T cell function, we systematically compared MHC class I and II expression within the TME of HPV α9 (HPV16-like) and HPV α7 (HPV18-like) CC. Our comparison found similar mRNA expression of genes involved in MHC class II antigen presentation between α9 and α7 HPV+ CC. Interestingly, RNA-seq analysis on genes for components of the MHC class I antigen presentation pathway identified a significant difference in the expression of several members of the PLC between α9 and α7 HPV. Specifically, the levels of *CANX*, *CALR*, and *PDIA3* mRNA expression in HPV α7 CC were comparable to that of HPV− CC. It is possible that viral integration status may be influencing these results, as HPV18 (α7) was seen to be integrated into all HPV+ CC within this cohort, whereas only 76% of HPV16 (α9)-related samples showed HPV integration [35]. In HPV16-related CC, downregulation of HLA-A, HLA-B, and HLA-C has been observed in CC with integrated HPV16 genomes, compared to those with episomal genomes [73]. HPV integration may influence the expression of MHC-related genes, contributing to the differences in MHC expression between the α7 and α9 CC cohorts. Overall, these differences in MHC I and II-related gene expression may affect antigen loading and perhaps contribute to the differential prognosis and overall survival observed between α9 (HPV16-like) and α7 (HPV18-like) CC.

MHC class II expression is particularly important in the anticancer immune response for the presentation of neoantigens. After their generation and programming in the thymus, CD8+ and CD4+ T cells circulate in the body until they encounter their specific antigen presented on MHC class I or class II molecules, respectively [15,74,75]. We found that both the classical and non-classical genes for MHC class II were expressed at higher levels in HPV+ CC compared to HPV− CC. *HLA-DQA1* was the only MHC II classical α- and β- chain gene that did not show significantly higher mRNA expression in the α7 CC cohort compared to HPV− CC cohort. This phenomenon may indicate that *HLA-DQA1* is a protective allele against certain HPV types. Several studies have previously shown associations between HLA alleles and cervical cancer, but not with *HLA-DQA1* [76,77,78,79,80,81]. Interestingly, one genome-wide association study identified that the strongest signal of association with CC comes from rs9272143, which is located between *HLA-DRB1* and *HLA-DQA1* [82].

Most genes involved in class II antigen-presentation pathways were also upregulated in HPV+ CC. The increased expression of the master transcriptional regulator *CIITA* in HPV+ CC is fully consistent with the upregulation of MHC class II gene expression observed in these tumors (Figure 3). The RFX complex is known to assemble on MHC class II promoters in vivo even while the genes are not expressed, as CIITA is the rate-limiting step responsible for the activation of transcription [54]. Therefore, it is not surprising that we did not observe a change in expression for the *RFX* family of genes between HPV+ and HPV− CC tumors.

In response to T cell activation, CTLA-4, encoded by *CD152*, is expressed and can outcompete CD28 for binding CD80/86. By binding CD80/86, CTLA-4 attenuates the cellular response initiated by the interaction of antigen-presenting MHC II and the TCR [55]. We found that *CD152* was significantly upregulated in HPV+ CC tumors compared to HPV−, providing evidence of higher levels of T cell activation (Figure 4). In addition, HPV+ tumors also demonstrated generally higher levels of other inducible T cell survival molecules (Figure 4). These survival signals are required for proliferating T cells to persist and survive after antigen recognition and subsequent stimulation with co-stimulatory molecules [55]. These data, supported by the higher expression of CD3-encoding genes, indirectly indicate that a higher number of activated and proliferating infiltrating T cells are present within the TME of HPV+ CC compared to HPV− CC (Figure S2).

Although MHC class II is predominantly produced in professional APCs, it can also be presented on amateur APCs, such as epithelial cells, in a proinflammatory environment [83]. Our data show that the expression of MHC class II-related genes appears to be several orders of magnitude above the markers for several types of professional APCs, including DCs (*CCL13*) and B cells (*CD19*). In comparison, the normalized read levels of MHC class II gene expression appeared comparable to that of epithelial (*CDH1*) and macrophage (*CD68*, *CD163*) specific genes. Although mRNA expression does not directly translate to protein levels within the tumor, numerous studies using immunohistochemistry (IHC) to investigate MHC class II expression in CC have conclusively shown high expression of these heavy chain proteins and proteins involved in MHC class II antigen presentation by the carcinoma cells [84,85,86,87,88]. These findings support our interpretation that epithelial cells within the actual tumor are coordinately expressing high levels of MHC class II genes, and potentially serving as amateur APCs in this moderately inflamed TME [83]. The high expression of *CD68* may also indicate that macrophages play a role in MHC class II expression within the TME of CC, although the significance of this contribution is unknown. *CD68* is upregulated in a proinflammatory environment, particularly by IFNγ which was seen to be expressed at higher levels in HPV+ CC (Figure 3). Furthermore, previous studies using single-cell RNA-seq show that macrophage infiltration into CC tumors is relatively low compared to other cell types, such as T and NK cells [89]. These findings indicate that while both macrophages and epithelial cells may be contributing to MHC class II expression in the CC TME, the bulk of their expression is provided by the far more prevalent epithelial-derived tumor cells, as reported by IHC studies.

Upon detection and binding their cognate antigen in the context of MHC class I, CTLs can kill virus-infected or neoplastic cells via secretion of the death-inducing granules: granzymes, perforin, cathepsin C, and granulysin [90]. We found that both the classical and non-classical genes for MHC class I were expressed at higher levels in HPV+ CC compared to HPV− CC (Figure 5). Genes involved in antigen processing were also generally increased in HPV+ CC, particularly within the HPV α9 cohort (Figure 6). *IFNG* expression also appeared higher in HPV+ CC, which is known to upregulate MHC class I expression (Figure 3). Some studies have observed high expression of MHC class I heavy chain proteins using IHC in CC, while others have identified a reduction in classical MHC class I heavy chain proteins as the disease progressed, likely due to mutational events [91,92].

For the establishment of a persistent infection, high-risk HPV types are known to transcriptionally downregulate MHC I heavy chain and other components necessary for antigen loading and presentation through their E7 oncoprotein [22,58,59]. MHC I is also downregulated using a non-transcriptional mechanism through the high-risk HPV E5 oncoprotein, in which the classical heavy chains are trapped in the Golgi apparatus and not transported to the cell surface [93]. Despite the immune evasion capabilities of the virus, our data may suggest that there is a higher expression of MHC class I within the TME of HPV+ CC, leading to higher CTL activity in these cancers. As our analysis addressed MHC I gene expression at the transcriptional level, our results do not rule out the possibility that other post-transcriptional mechanisms can reduce antigen presentation. However, IHC detection of MHC I proteins indicated that only a subset of approximately 40% of HPV+ CC have reduced expression of one or more MHC I gene components at the protein level [94]. As the samples in this study are carcinomas, it is also possible that E7 could reduce MHC I transcription in non-cancerous or pre-cancerous lesions, although the reacquisition of MHC I expression during cancer progression is difficult to reconcile with current models of tumorigenesis. These differences in MHC I expression in HPV+ cancers compared to HPV infection have been previously highlighted through an analysis of the TCGA HNSCC and CC cohorts [27].

Higher *IFNG* expression within HPV+ CC may also contribute to the increased neoantigen presentation in the context of MHC through the activity of APOBEC3. IFNγ can induce the activity of the cytosine deaminase APOBEC3, a mechanism of innate defense against exogenous viruses and endogenous retroelements [65]. APOBEC3 has also demonstrated the ability to induce tumor mutations through aberrant genomic DNA editing mechanisms [64]. APOBEC-mediated mutations appear to occur at a later stage of tumor evolution, and the rate of APOBEC mutation has been positively correlated with increased expression of mutation-induced neoantigens, which may drive the host immune response [95]. Despite the higher expression of *APOBEC3A*, *3B*, and *3H* in HPV+ CC compared to HPV− CC (Figure S3), there appears to be a higher level of predicted neoantigens in HPV− CC, indicating that these neoantigens may not be induced through the activity of APOBEC3 [29]. Therefore, it is still unclear if and how the higher expression of APOBEC3-related genes, likely induced by the proinflammatory environment within HPV+ CC, may change the tumor microenvironment of these cancers compared to HPV− CC.

The observed increase in the expression of both MHC class I and II-related genes in HPV+ CC indicate that these tumors display a more immune “hot” phenotype [96]. This phenotype is consistent with the detection of higher CD4+ and CD8+ T cell activation within the TME and is further supported by our previous findings that HPV+ CC have greatly increased lymphocyte infiltration and higher expression of activation/exhaustion markers [29]. Conversely, HPV− CC demonstrate an immune “cold” phenotype with lower T cell activation and reduced MHC-related gene expression [29]. Although limited by the small sample size for HPV− CC, these different phenotypes may influence treatment approaches to the distinct tumor types, particularly for immunotherapies. Immune checkpoint inhibitor therapies, such as anti-PD-1, are a form of immunotherapy that introduce monoclonal antibodies targeting immune inhibitory receptors. By targeting inhibitory receptors, these therapies enhance the efficacy of antitumor immune responses, revitalize exhausted CTLs for tumor cell killing, and have greatly improved the clinical outcomes of many cancers [97]. Studies using IHC and RNA-seq have identified correlations between MHC class II expression on tumor cells and disease-free progression with a positive response to immunotherapies such as anti-PD-1 [67,98,99,100,101,102,103,104]. Furthermore, loss of MHC class I expression has been suggested as a predictor for the development of resistance to immune checkpoint inhibitor therapies in HPV−associated CC, as well as other cancers [105,106,107,108]. As these therapies rely on CTLs and the expression of immune checkpoint markers, which are upregulated through inflammatory signaling, the observed differences between HPV+ and HPV− CC, as well as subtle differences between HPV α9 and α7 CC, suggest that these tumors may not respond equally to immune checkpoint inhibition.

Due to the important role of the T cell response in immune checkpoint inhibitor therapy, as well as the recent approval of these drugs for PD-L1-positive CC, it will likely be important to understand the consequences of these differences in antigen presentation capacity on their efficacy in these immunologically distinct cancers. Currently, clinical trials investigating immunotherapies and their contributions to treatment response in CC do not consider HPV status or HPV type, including studies for an anti-PD-1 monoclonal antibody pembrolizumab [109,110,111,112,113,114,115,116,117]. Our identification of these significant differences in MHC class I and II expression between HPV+ and HPV− CC highlights the need for patient stratification based on HPV status in clinical trials to determine its impact on responsiveness to various anti-cancer therapies.

## Data Availability

Not applicable.

## Supplementary Materials

The following supporting information can be downloaded at: https://www.mdpi.com/article/10.3390/cells11233911/s1, Figure S1: Expression of cell surface marker genes for APCs and epithelial cells in CC stratified by HPV+ (α9 or α7) and HPV− status; Figure S2: Expression of cell surface marker genes for IFNγ-producing lymphocytes in CC stratified by HPV+ (α9 or α7) and HPV− status. Figure S3: Expression of the *APOBEC3A*, *3B*, and *3H* genes in CC, stratified by HPV+ (α9 or α7) and HPV− status. Table S1: HPV Annotation of TCGA CESC Samples.
