# Supplementary material for: Reduced MHC Class I and II Expression in HPV−Negative vs. HPV−Positive Cervical Cancers
Source: Cells. 2022 Dec 3;11(23):3911. doi: 10.3390/cells11233911 (PMC9741043; doi:10.3390/cells11233911)
Supplement: Supplementary file 1 [file cells-11-03911-s001.zip › Supplementary Figures.pdf]

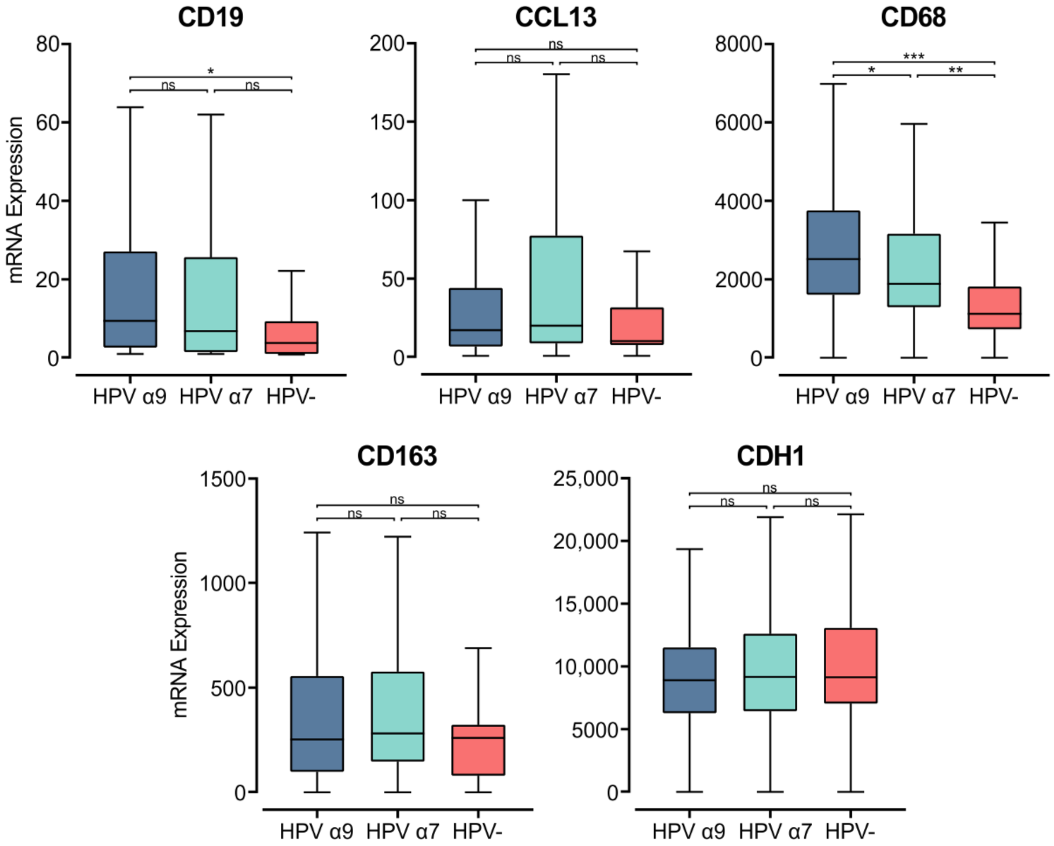

Figure S1: Expression of cell surface marker genes for APCs and epithelial cells in CC stratified by HPV+ (α9 or α7) and HPV- status. Normalized RNA-seq data for B cells, dendritic cells, macrophages, and epithelial cells were extracted from the CC cohort of the TCGA database. *** p ≤ 0.001, ** p ≤ 0.01, * p = 0.05, ns (not significant).

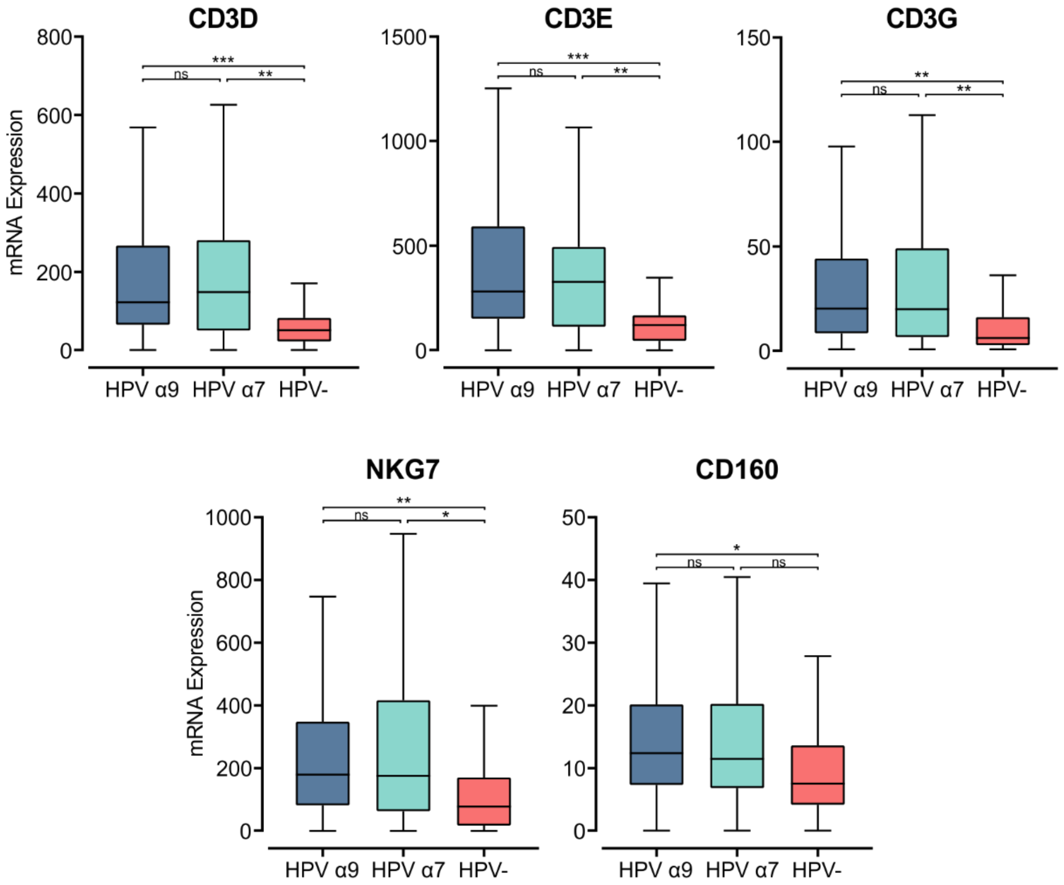

Figure S2: Expression of cell surface marker genes for IFNγ-producing lymphocytes in CC stratified by HPV+ (α9 or α7) and HPV- status. Normalized RNA-seq data for T cells and NK cells were extracted from the CC cohort of the TCGA database. *** $p \leq 0.001$, ** $p \leq 0.01$, * $p = 0.05$, ns (not significant).

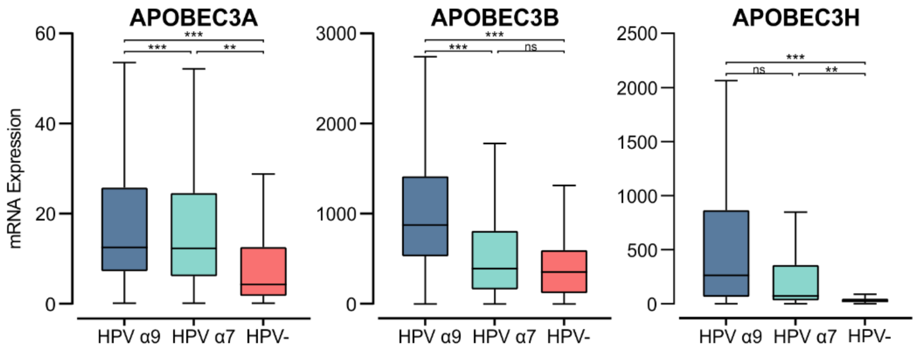

Figure S3: Expression of the *APOBEC 3A*, *3B*, and *3H* genes in CC, stratified by HPV+ (α9 or α7) and HPV- status. Normalized RNA-seq data for these genes were extracted from the CC cohort of the TCGA database. *** p ≤ 0.001, ** p ≤ 0.01, * p = 0.05, ns (not significant).
